# Asymmetrical Training Scheme of Binary-Memristor-Crossbar-Based Neural Networks for Energy-Efficient Edge-Computing Nanoscale Systems

**DOI:** 10.3390/mi10020141

**Published:** 2019-02-20

**Authors:** Khoa Van Pham, Son Bao Tran, Tien Van Nguyen, Kyeong-Sik Min

**Affiliations:** Electrical Engineering Department, Kookmin University, Seoul 02707, Korea; khoapv@kookmin.ac.kr (K.V.P.); sontb@kookmin.ac.kr (S.B.T.); tiennv@kookmin.ac.kr (T.V.N.)

**Keywords:** asymmetrical training scheme, binary memristor crossbar, neural networks, energy-efficient edge computing, nanoscale systems

## Abstract

For realizing neural networks with binary memristor crossbars, memristors should be programmed by high-resistance state (HRS) and low-resistance state (LRS), according to the training algorithms like backpropagation. Unfortunately, it takes a very long time and consumes a large amount of power in training the memristor crossbar, because the program-verify scheme of memristor-programming is based on the incremental programming pulses, where many programming and verifying pulses are repeated until the target conductance. Thus, this reduces the programming time and power is very essential for energy-efficient and fast training of memristor networks. In this paper, we compared four different programming schemes, which are F-F, C-F, F-C, and C-C, respectively. C-C means both HRS and LRS are coarse-programmed. C-F has the coarse-programmed HRS and fine LRS, respectively. F-C is vice versa of C-F. In F-F, both HRS and LRS are fine-programmed. Comparing the error-energy products among the four schemes, C-F shows the minimum error with the minimum energy consumption. The asymmetrical coarse HRS and fine LRS can reduce the time and energy during the crossbar training significantly, because only LRS is fine-programmed. Moreover, the asymmetrical C-F can maintain the network’s error as small as F-F, which is due to the coarse-programmed HRS that slightly degrades the error.

## 1. Introduction

The increase in the number of edge devices such as mobile devices, smart phones, etc. demand a larger amount of data processing with higher energy efficiency. Edge computing is defined as the method that can move the control of data processing from the high-performance cloud systems to the last-edge devices of Internet of Things such as smart sensors, etc., where various data are sensed, collected, and generated from the physical environment. Currently, most data collected from the physical world are unstructured data such as images, voices, anomaly patterns, etc., [1,2]. In order to interpret a vast amount of unstructured data from the physical world, neural network techniques such as deep learning should be embedded in the edge-computing devices. If we try to process all these unstructured data only by the cloud systems, the power consumption of cloud servers and data centers may jump up to an uncontrollable level. Thus, it is widely expected that the edge computing technique may be in the mainstream in 2 to 5 years, as neural-network-based deep learning, Internet of Things, and smart sensors contribute to each other mutually to advance the edge-computing technique further [3,4].

**Figure 1:**
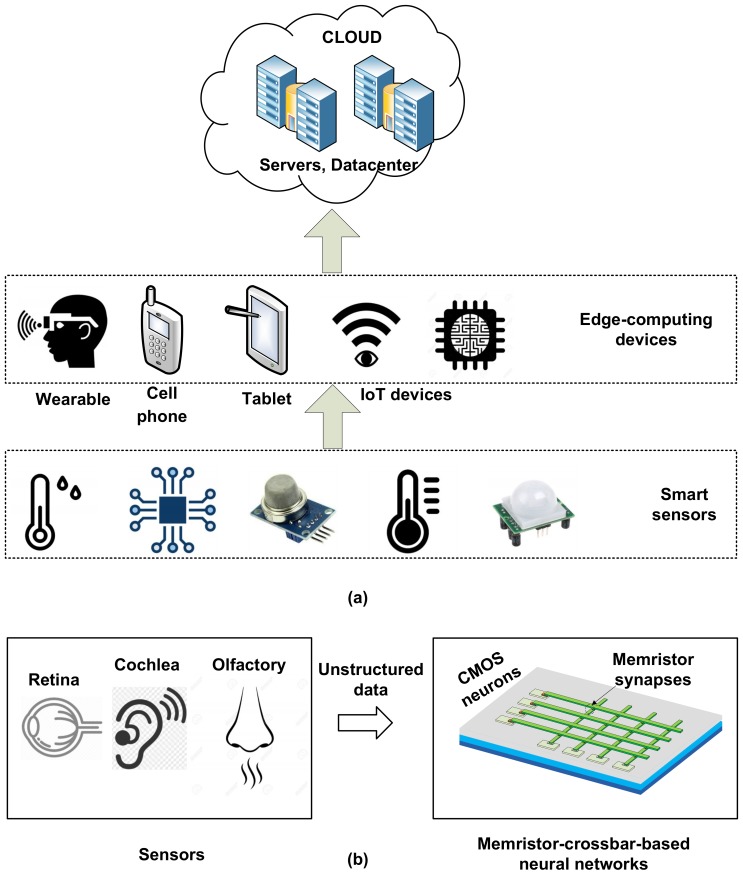
(**a**) The conceptual diagram of cloud, edge-computing devices, and sensors. (**b**) The block diagram of an edge-computing device composed of memristor-crossbar-based neural networks for processing the unstructured data.

The memristor-based neural networks can be integrated to the edge-computing nanoscale systems easily. The memristive networks are inspired from the biological concepts of human brain processing that has been developed to replace the conventional Von Neumann computing architecture in the future [5,6,7,8]. For implementing the non-Von-Neumann computing architecture, memristors can provide various advantages of scalability, low-energy consumption, non-volatility, and potential 3-dimensional stacking [9,10,11] since its first experimental demonstration [12]. Figure 1a shows the conceptual diagram of the cloud systems, edge-computing devices, and Internet-of-Things (IoT) sensors [13,14,15]. Figure 1b shows the block diagram of the edge-computing device composed of memristor-crossbar-based neural networks.

The sensors in the conceptual diagram collect a huge amount of unstructured data from the physical environment, as shown in Figure 1a. The increased demand of the information processing of the huge amount of unstructured data makes the memristive neural networks useful in future edge-computing applications [16,17]. The unstructured data can be delivered to the next edge devices such as smartphones, or they can be directly connected to the cloud servers for higher-level data processing. The cloud systems are the final destination of all these data, where they are stored, interpreted, re-organized, and understood intelligently to make them useful in human life [13,14,15]. Thus, the memristive neural networks are needed to process the unstructured data at the edge instead of transferring the physical-world data directly to the cloud systems [17]. The memristor-crossbar-based neural networks can be very useful in these various edge-computing applications, where low-energy consumption is needed [17]. Saving the training energy and training time of memristor-crossbar networks can be very useful in the nanoscale edge-computing systems.

For developing memristor-based neural networks, we can start from both analog and binary memristors. First, for analog memristors, they were observed to show a gradual change in memristor’s conductance, according to the electrical pulses applied [18,19,20]. They can be used as analog memories in neuromorphic applications. However, unlike the analog memristors, the other memristors show the switching behaviors that seem abrupt and sharp between the High Resistance State (HRS) and the Low Resistance State (LRS) instead of changing gradually between two states [21,22,23,24,25]. They are switched just from LRS to HRS and vice versa and do not allow any intermediate states between LRS and HRS for the applied voltage or current pulses. In this study, LRS and HRS mean a Low Resistance State and a High Resistance State, respectively. Since many memristors are based on the filamentary conduction mechanism with the abrupt transition between LRS and HRS [26], in this paper, we focus on binary memristor crossbars. In the binary memristors, only HRS and LRS are used in programming. One advantage of binary crossbars is that binary states of HRS and LRS can be programmed more reliably than the intermediate states between HRS and LRS. For both analog and binary memristors, a selector such as transistor can be used to reduce the disturbance problems in read and write operations like the sneak leakage [27,28,29].

**Figure 2:**
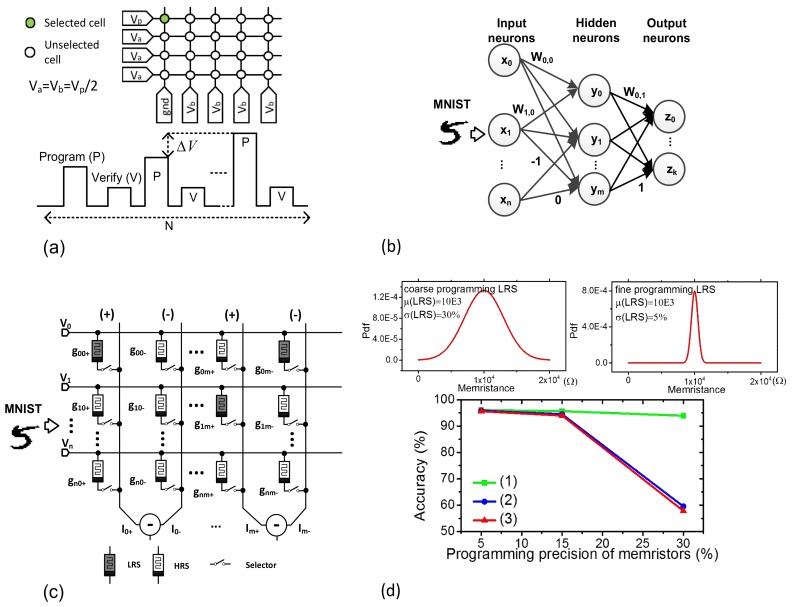
(**a**) The program-verify scheme for programming memristors. (**b**) The diagram of multiple-layer neural networks. (**c**) The schematic of binary-memristor-based networks. (**d**) The memristance distributions on Low Resistance State (LRS) cells with the percentage σ = 30% and 5% are shown in the upper region, respectively. σ = 30% and σ = 5% mean the memristor programing precision is coarse and fine, respectively. ‘pdf’ means the probability density function. The network accuracy of Modified National Institute of Standards and Technology (MNIST) data-set while varying the programming precision is shown in the lower. (1) The programming precision of HRS is changed from 5% to 30%, while maintaining the LRS precision is 5%. (2) The programming precision of LRS is changed from 5% to 30%, while maintaining the HRS precision at 5%. (3) The programming precision of both HRS and LRS is changed from 5% to 30%.

For realizing neural networks with binary memristor crossbars, memristors should be programmed by HRS and LRS, according to the training algorithms such as the backpropagation [30]. Unfortunately, it takes a very long time and consumes a large amount of power in training memristor crossbars because the program-verify scheme such as Incremental Step Pulse Programming (ISPP) is based on the incremental pulse steps [31]. Thus, reducing the programming time and power of memristors is very essential for energy-efficient and fast training of memristor neural networks. Figure 2a shows the program-verify scheme of memristors, where the pulse amplitude is modulated gradually, according to the number of programming pulses. After the programming pulse, the verifying pulse follows. Here ΔV and N mean the incremental voltage step and the number of programming pulses applied to memristors, respectively.

To explain the memristor-crossbar-realized networks in detail, see Figure 2b, which is composed of the input, hidden, and output neurons represented with x_i_, y_i_, and z_i_, respectively. In this case, i means the neuron index. We assumed the numbers of input, hidden, and output neurons are n, m, and k, respectively. W_0,0_ W_1,0_, etc. are the synaptic weights for layer #0. W_0,1_ and W_1,1_, etc. are for layer #1. The synaptic weights are represented with only −1, 0, and 1, in the binary-crossbar-based networks. The binary-crossbar-based networks can be thought one of the ternary neural networks that were proposed to solve the computational overloading problem of full-precision neural networks. The full-precision networks are known to demand very large amounts of computation with floating-point numbers [32,33]. In the ternary networks, the high-precision multiplication can be replaced with the simple bitwise operation [34,35]. By doing so, the computing power and hardware complexity can be greatly reduced in the ternary networks, in spite of some loss of accuracy, compared to the high-precision neural networks [34].

The schematic of binary-memristor-based networks is shown in Figure 2c. In this case, HRS and LRS are represented as white and gray, respectively. In Figure 2c, the memristors are connected to the selectors. In this scenario, (+) and (−) columns mean the positive and negative columns, respectively [36]. The input signals are applied to the rows of the crossbar. For the first (+) and (−) columns and the first row of V_0_, we can calculate the column current of (g_00+_ − g_00−_) V_0_. If both g_00+_ and g_00−_ are HRS, the calculated (g_00+_ − g_00−_) V_0_ is regarded zero. If g_00+_ and g_00−_ are LRS and HRS, respectively, the column current becomes positive. If they are HRS and LRS, the current becomes negative. By doing so, we can represent 0, +1, and −1 with (g_00+_ = HRS, g_00−_ = HRS), (g_00+_ = LRS, g_00−_ = HRS), and (g_00+_ = HRS, g_00−_ = LRS), respectively.

For simulating the binary memristor crossbar in Figure 2c, first, we assume that the memristors programmed during the training time have Gaussian statistical distribution, as shown in the upper region in Figure 2d. The variance of the Gaussian distribution function, σ^2^ can be affected by the programming precision of memristors. If the memristors are programmed with fine precision, the memristance distribution becomes more narrow, which results in a smaller value of σ. On the contrary, the coarse programming can increase σ larger in the memristance distribution, as shown in Figure 2d. The upper region in Figure 2d shows the distributions of memristance with the percentage σ = 30% and 5%, respectively. σ = 30% and σ = 5% mean the corresponding precision of memristor programming is coarse and fine, respectively. The network’s accuracy was tested with Modified National Institute of Standards and Technology (MNIST) data-set [37] with a few cases of HRS and LRS programming precisions, as shown in the lower region in Figure 2d. In this study, we simulated three cases. (1) The programming precision of HRS is changed from 5% to 30% while maintaining the LRS precision at 5%. (2) The programming precision of LRS is changed from 5% to 30%, while maintaining the HRS precision at 5%. (3) The programming precision of both HRS and LRS is changed from 5% to 30%. The lower region in Figure 2d indicates clearly that the network’s accuracy is more affected by the programming precision of LRS than the precision of HRS.

Based on this asymmetrical effect of HRS and LRS programming on the network’s accuracy, in this paper, we propose a new asymmetrical scheme of the fine-LRS and coarse-HRS programming. The proposed programming scheme can minimize the programming time and power, while maintaining the network’s accuracy as high as possible. In the following section, we analyze how the programming precision is related to the programming time, power, and the network’s accuracy. Based on the analysis, we verify that the proposed asymmetrical programming scheme is very energy-efficient and fast in training the binary-memristor-crossbar networks. This memristor-crossbar-based network can be very useful in the edge-computing nanoscale systems, which demand the low-energy consumption desperately to process the vast amount of unstructured data from the physical environment.

## 2. Materials and Methods

**Figure 3:**
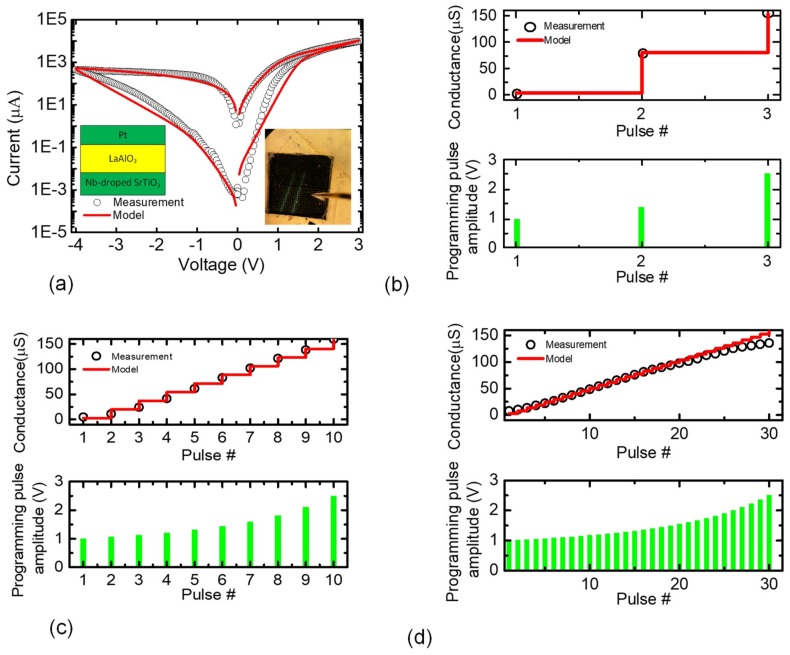
(**a**) The measured and calculated current-voltage relationships of the fabricated memristor, the cross-sectional view of the measured device, and the microscope picture of the measured device. (**b**) The coarse programming scheme and the memristor’s conductance changes. (**c**) The moderate programming scheme with the memristor’s conductance changes. (**d**) The fine programming scheme and the memristor’s conductance changes.

Figure 3a shows the measured and calculated current-voltage relationship of the fabricated memristors. The open-circle symbol and the red line in Figure 3 represent the measured data and its behavioral model, respectively. The mathematical model equations of the measured memristive behavior can be found in the previous publication [16]. The equations were implemented in Verilog-A in the CADENCE SPECTRE (Cadence Design Systems, Inc., San Jose, CA, USA) for the hybrid circuit simulation of memristors and complementary metal-oxide-semiconductor (CMOS) circuits. The cross-sectional view is also shown in the inset of Figure 3a, where the device has the film structure of the Pt/LaAlO_3_/Nb-doped SrTiO stacked layer [38]. The microscope picture of the measured device is also shown in Figure 3a, where the top electrode area is 100 μm × 100 μm. The top and bottom electrodes were formed by Platinum (Pt) and SrTiO_3_, respectively [38]. The measurement was performed with Keithley-4200 (Semiconductor Characterization System, Tektronix, Inc., Beaverton, OR, USA) combined with the Keithley-3706 Switching Matrix. To program the memristor device, we used the program-verify scheme with the new pulse-amplitude modulation [16]. Figure 3b shows the coarse programming scheme with the memristor’s conductance change and the applied programming pulses. In this case, we changed the pulse amplitude very coarsely, as shown in the lower region in Figure 3b. The number of programming pulses modulated in Figure 3b is just as small as three for the coarse programming scheme. To do so, the average ΔV per pulse was adjusted as large as 0.75 V and the average conductance change per pulse was measured as large as 67 μS. In this case, the target conductance values of LRS and HRS are assumed to be 105 μS and 2 μS, respectively. The measured HRS/LRS ratio is ~50. The measured conductance can be calculated with the mathematical model equations in Reference [25]. In Figure 3b, the red line shows the programmed conductance calculated by the model equations in Reference [25]. Figure 3c shows the moderate programming scheme, where we increased the number of programming pulses from three of the coarse programming to 10 of the moderate scheme. The average ΔV per pulse is reduced from 0.75 V to 0.34 V for the moderate scheme. By doing so, in Figure 3c, the average conductance change is decreased from 67 μS to 15 μS. Figure 3d shows the fine programming scheme, where the number of programming pulses is as large as 30. In this case, the average ΔV per pulse should be as small as 0.05 V. We can adjust the memristor’s conductance as fine as 4.4 μS on average for each programming pulse, as shown in Figure 3d. In Figure 3c,d, the open circle and the red line represent the measured and the calculated conductance, respectively, like Figure 3b. From Figure 3b–d, we can see that the measured conductance values are in good agreement with the calculation, for the coarse, moderate, and fine programming schemes, respectively.

To analyze how the programming precision is related to the programming time and power, we compared the coarse, moderate, and fine programming schemes in this paper. For the programming time, the coarse, moderate, and fine schemes need 3, 10, and 30 programming pulses, respectively, for changing memristance from HRS to LRS. Similarly, we can calculate the energy consumption for the three programming schemes during the training time. From the circuit simulation with the Verilog-A model of memristors measured in Figure 3a, we can calculate the memristor’s current response changed dynamically with respect to the programming time, according to the applied programming voltage pulse. The programming energy can be calculated by integrating the product of the applied voltage pulse and the memristor’s current response over the programming time. In this study, the CMOS programming circuit was not considered in calculating the programming energy because the power consumption of CMOS programming circuit was estimated to be much smaller than the power consumption in the memristor array. The calculated programming energy of the coarse and fine schemes are 680 nJ and 1600 nJ, respectively, for programming the memristor from HRS to LRS. As expected, the fine scheme needs 2.4× larger energy and 10× longer time in HRS-to-LRS programming than the coarse one. On the contrary, for the programming precision, the fine scheme can adjust the conductance change, ΔG, which is as precise as 4.4 μS, compared to ΔG = 67 μS of the coarse programming.

## 3. Results

**Figure 4:**
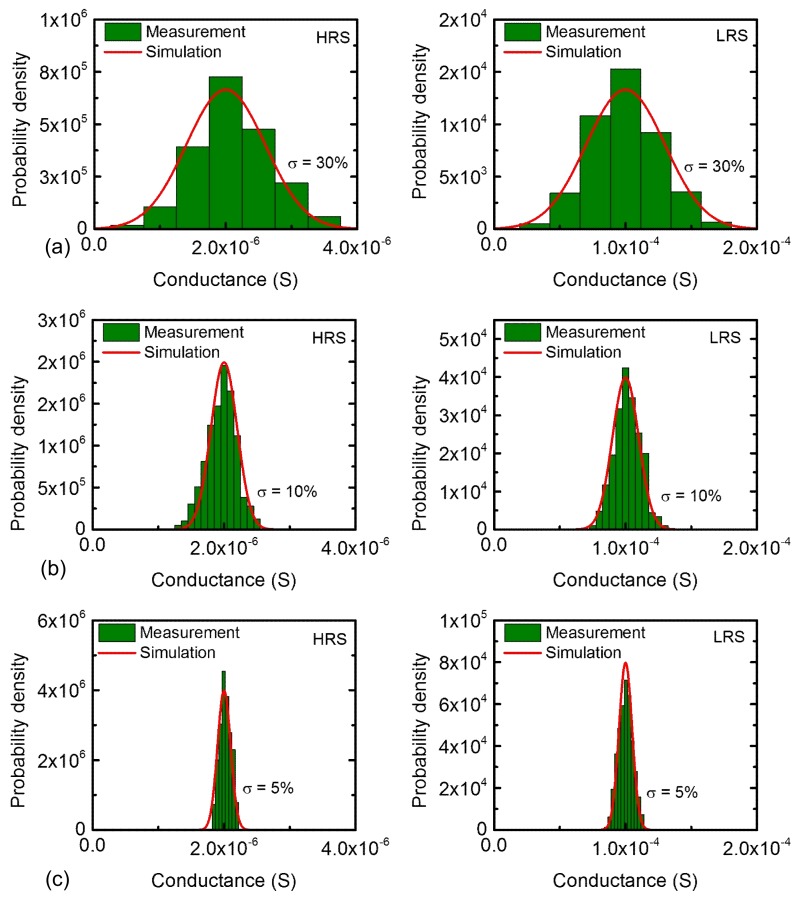
The memristance distributions of HRS and LRS for (**a**) the coarse programming, (**b**) the moderate programming, and (**c**) the fine programming schemes, respectively.

Figure 4a shows the measured and calculated distributions of HRS and LRS, respectively, which are programmed by the coarse scheme in Figure 3b. In this scenario, the percentage σ was measured around 30% for both HRS and LRS distributions. In Figure 4a, the red line represents the calculated probability density function and the columns are obtained from the measured probability density of memristor’s conductance. From this figure, we can see the measurement agrees well with the calculated probability density function. Similarly, we trained memristors with the moderate and fine programming schemes of Figure 3c,d, respectively. Using the moderate and fine training, the narrower distributions of programmed HRS and LRS are obtained, as shown in Figure 4b,c, respectively. In this scenario, we measured σ as small as 10% and 5% for Figure 4b,c, respectively. Combining Figure 4a–c, the percentage σ values of 30%, 10%, and 5% are obtained from the measured memristor’s conductance with the coarse, moderate, and fine programming schemes, respectively. In addition, we can program memristor crossbars with a very fine or very coarse distribution such as σ = 1% or σ = 50%. If the lowest error rate is required, we should try to program the crossbar with a very tight distribution such as σ = 1%. Yet, if the error rate can be very bad, we can program the crossbar as coarse as σ = 50%. By doing so, we can use the trade-off relationship of the error rate and the training energy to optimize the network performance and energy in a given circumstance. However, in terms of real programming, it is very difficult to control the conductance distribution within σ = 1%. For σ = 1%, the conductance change per pulse should be controlled within ~1 μS. This fine resolution of conductance change needs an infinitely large number of programming pulses, a very small ΔV, and a very precise control of voltage amplitude modulation. Thus, this fine resolution within ~1 μS cannot be realized in the real programming circuit. In this paper, considering the practical programming circuit, we chose σ = 5%, σ = 10%, and σ = 30%, for three schemes of memristor programming. Particularly, the σ as small as 5% of the fine scheme is very similar to the standard deviation of memristor’s conductance programmed in the training of the analog memristor-based neural network [29]. In addition, for a very coarse distribution such as σ = 50%, the number of pulses can be just one or two. If so, some high conductance values of HRS distribution can be overlapped with some low conductance values of LRS. Thus, to avoid the overlap between HRS and LRS distributions, we limited the σ to less than 30% in this paper.

**Figure 5:**
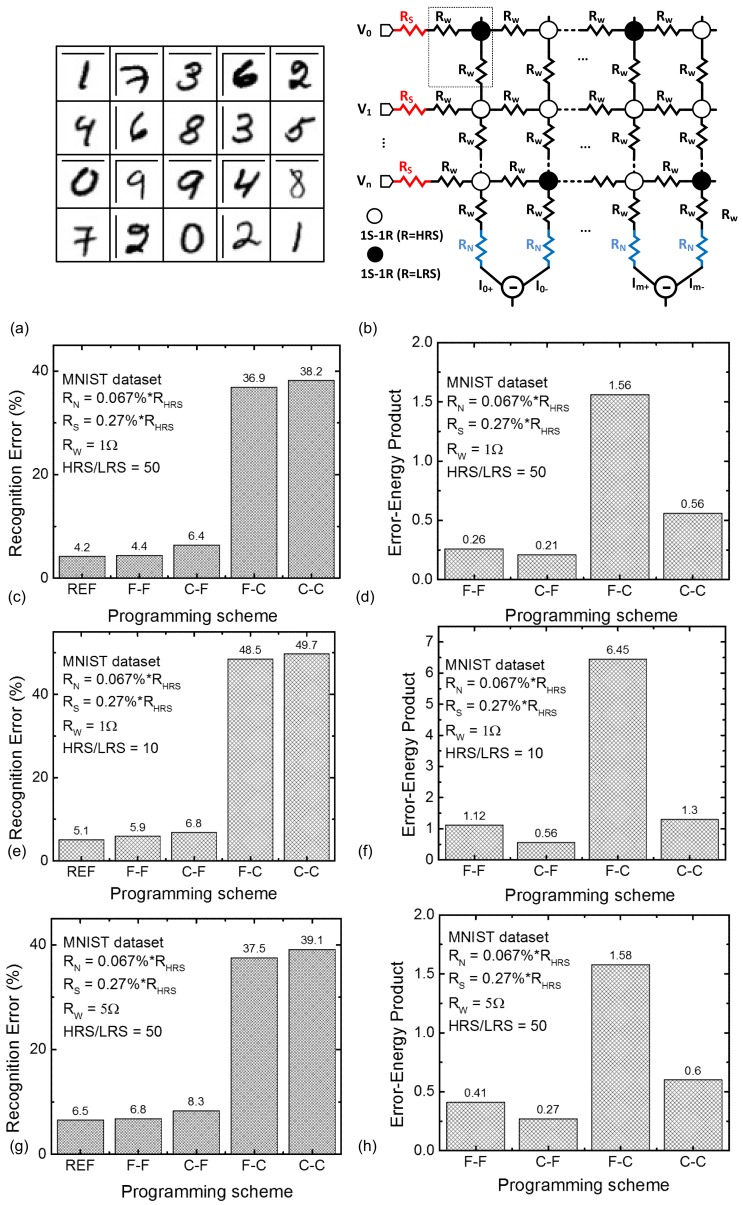
(**a**) The MNIST vectors. (**b**) The binary-memristor crossbar with R_S_, R_N_, and R_W_. (**c**) The recognition error with HRS/LRS = 50 and R_W_ = 1 Ω. (**d**) The error-energy product with the HRS/LRS ratio = 50 and R_W_ = 1 Ω. (**e**) The recognition error with HRS/LRS = 10 and R_W_ = 1 Ω. (**f**) The error-energy product with HRS/LRS = 10 and R_W_ = 1 Ω. (**g**) The recognition rate with HRS/LRS = 50 and R_W_ = 5 Ω. (**h**) The error-energy product with HRS/LRS = 50 and R_W_ = 5 Ω.

As mentioned in Figure 3, HRS and LRS can be trained by various combinations of the coarse, moderate, and fine programming schemes. In this paper, we compared various combinations of programming schemes by testing the MNIST recognition error. The MNIST vectors of the hand-written digits of 0–9 are shown in Figure 5a. In this paper, we used 60,000 MNIST vectors for training the binary-memristor-based crossbar. After training the crossbar, we tested 10,000 MNIST execution vectors for evaluating the recognition error with different programming schemes. 

The MNIST recognition rate is simulated with a SPICE-like framework implemented in the MATLAB software (MathWorks, Inc., Natick, MA, USA) [39]. In this case, we considered various non-ideal effects in the memristor crossbar such as source resistance, neuron resistance, and wire resistance, as shown in Figure 5b [39,40,41]. In Figure 5b, R_S,_ R_N,_ and R_W_ mean the parasitic source, neuron resistance, [39] and wire resistance, [40,41] respectively. In the simulation, we assumed R_S_ = 0.27% of R_HRS_, R_N_ = 0.067% of R_HRS_, R_W_ = 1–5 Ω, respectively. These parasitic resistance values were obtained by measuring the fabricated crossbar [39,40]. The MATLAB simulation results were also verified with the CADENCE circuit simulation [42]. In the MATLAB simulation, first, a netlist of memristor synapses, voltage sources, source resistance, neuron resistance, and wire resistance are generated as shown in Figure 5b [39]. In addition, the generated netlist is solved by MATLAB to calculate the crossbar’s column current. The simulation steps used in the paper are the same as the previous publication [39]. For training the synaptic weights, the conventional backpropagation algorithm was implemented in the MATLAB simulation [39].

In Figure 5c, REF represents the crossbar programmed with the ideal σ(HRS) = 0% and σ(LRS) = 0%. The zero σ means the conductance values of HRS and LRS are fixed by 2 μS and 100 μS, respectively. Because this zero σ could not happen in programming real crossbars, we assumed the crossbar programmed with zero σ as the reference value in this paper. From the measurement, the HRS/LRS ratio was observed to be around 50, as measured in Figure 3. In Figure 5c, C-C means both HRS and LRS are programmed by the coarse scheme with a low precision of memristor programming. C-F means HRS and LRS are programmed by the coarse and fine scheme, respectively. On the other hand, F-C is for HRS and LRS programmed by the fine and coarse, respectively. Lastly, F-F means both HRS and LRS are programmed by the fine scheme with high precision. As a proof of concept, even though a large amount of programming error is observed in the coarse programming scheme on HRS cells in C-F, the network’s error of C-F can be maintained as low as 6.4% compared to 4.4% of F-F, as shown in Figure 5c. The effect of the coarse-programming of HRS cells is negligible in terms of the network’s error. On the contrary, when both HRS and LRS cells are controlled with the coarse-programming scheme of C-C, the network’s error becomes as large as 36.9%. The narrow gap of the recognition error between F-F and C-F in Figure 5c clearly emphasizes that the programming precision of LRS cells is more important than the precision of HRS in terms of the network’s error. Thus, C-F can be more energy-efficient than F-F, where both HRS and LRS are programmed by the fine scheme. In C-F, HRS can be coarse-programmed to save the programming energy and time. To consider both the recognition error and the programming energy, we define a new metric of the ‘error-energy product’ in this paper, which is calculated with the following Equation (1).

(1) Error−Energy Product=Recognition error×Training energy

In this scenario, the recognition error is obtained from Figure 5c. The training energy can be calculated with the programming time and power of the binary-memristor crossbar during the training time for 60,000 MNIST vectors. By doing so, Figure 5d compares F-F, C-F, F-C, and C-C, in terms of the error-energy product defined in Equation (1). As expected, C-F shows the smallest error-energy product. It means that C-F has the best energy efficiency in recognizing MNIST vectors among the four schemes of F-F, C-F, F-C, and C-C.

For implementing the memristor-based network on large-size crossbars, the HRS/LRS ratio is very important. With a high HRS/LRS ratio, the network’s error can be improved. However, if the HRS/LRS ratio becomes smaller, the recognition error can be degraded to worse levels. Thus, we evaluated the recognition error when the HRS/LRS ratio is decreased from 50 to 10. In Figure 5e, we assumed the HRS/LRS ratio is as low as 10 and calculated the recognition error and the error-energy products of the four cases of coarse and fine schemes. Figure 5e shows the recognition errors of F-F, C-F, F-C, and C-C, with the HRS/LRS = 10. Figure 5f compares the error-energy products of F-F, C-F, F-C, and C-C, with the HRS/LRS = 10. In Figure 5f, C-F also shows the smallest error-energy product such as in Figure 5d.

Figure 5g shows the recognition errors of F-F, C-F, F-C, and C-C, when the wire resistance is increased from 1 Ω to 5 Ω and the HRS/LRS ratio is fixed by 50. In this case, F-F and C-F show the errors as low as 6.8% and 8.3%, respectively. The gap of the recognition error between F-F and C-F is as narrow as 1.5%. In Figure 5h, among F-F, C-F, F-C, and C-C, C-F shows the smallest error-energy product like the previous comparison cases.

## 4. Discussion

We have a couple of things to discuss in this paper. First, generally, the probability density of the measured memristance distribution was known as the log-normal function instead of the Gaussian one [43]. However, it should be noted that the log-normal distribution of memristance was observed only when a single programming pulse was applied to the memristor instead of using the program-verify scheme shown in Figure 2a [43]. In this paper, we used the program-verify scheme with the pulse-amplitude modulation for programming memristors instead of the single-pulse programming. In the program-verify scheme, a programming pulse is generated first and then the conductance measured by the verifying pulse is compared with the target value. According to the measured difference with the target, the following programming pulse is generated to decrease or increase the conductance. In this program-verify scheme, the conductance measured in this paper was observed to have Gaussian distribution instead of the log-normal. The Gaussian distribution observed in this paper was also mentioned in the recent experiment of the HfO_2_-based memristor array [29]. The Gaussian distribution function can also be found in the threshold voltage of flash memory cells programmed by the program-verify scheme such as ISPP [44,45].

The second discussion is that the number of programming pulses and the interval time may affect the retention time of programmed HRS and LRS. As stated earlier, the coarse scheme has a smaller number of programming pulses than the fine scheme. Thus, if the retention time of the coarse scheme is shorter than that of the fine scheme, the neural network’s performance can be degraded more by the shorter retention time of the coarse scheme. This can make it very difficult to estimate the network’s performance accurately. To maintain the network’s performance constant, we should recover the synaptic weights to the originally-programmed values within the retention time. In this paper, we performed the analysis on the retention time of the measured memristors, as explained below. Based on the analysis, it has been verified that the retention time of the coarse scheme is almost the same with that of the fine scheme. It means that the network’s performance can be maintained at an almost constant rate, if the crossbar is reprogrammed within the retention time.

The previous publications reported that the number of programming pulses, and the interval time may affect the retention loss in some memristors such as WO_x_ [46]. Quantitatively, the retention loss can be modeled with the stretched-exponential Equation (2) [46,47,48].

(2) $$\varphi \left(t\right)=\varphi_{0}\exp\;(-{\left(\frac{t}{\tau }\right)}^{\beta })$$

**Figure 6:**
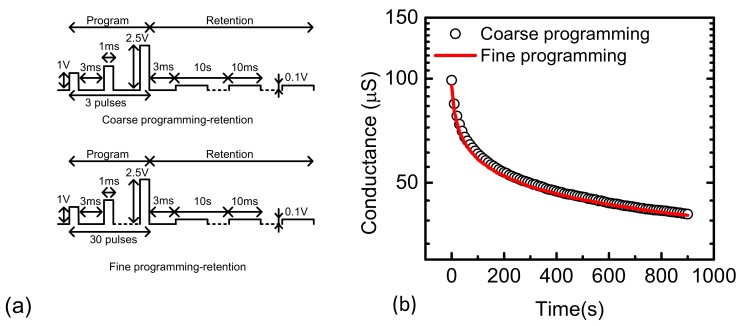
(**a**) The coarse and fine programming schemes showing the pulse width and interval time. (**b**) The LRS retention loss from 0 s to 900 s with the coarse and fine programming schemes.

Here φ(t) is the relaxation function, τ is the characteristic relaxation time, φ_0_ is the pre-factor, and β is the stretch index ranging between 0 and 1 [46]. In this case, we extracted τ and β of LRS retention loss for the fine and coarse programming, respectively. Figure 6a shows the coarse and fine programming schemes. For the coarse scheme, the number of programming pulses is only 3 and the pulse width and interval time are 1 ms and 3 ms, respectively. For the fine scheme, the number of programming pulses applied to the memristor is as many as 30. The pulse width and interval time are the same with the coarse scheme. Figure 6b shows the LRS retention loss from 0 s to 900 s with the coarse and fine programming schemes, which are represented by the circle symbol and red line, respectively. From this figure, β was found to be 0.26 for both the coarse and fine schemes. The extracted relaxation time, τ, is 1400 s and 1464 s, for the coarse and fine schemes, respectively. While the fine scheme has a relaxation time that is slightly longer than the coarse one, the difference in τ between the coarse and fine schemes seems very small. Similarly, the HRS retention loss also shows very little discrepancy between the coarse and fine schemes. Thus, in this paper, we can ignore the retention loss problem that may be caused by the different numbers of programming pulses of coarse and fine schemes.

The memristance statistical distributions in Figure 4 caused from device-to-device variation are obtained from the memristors fabricated as an isolated device instead of a crossbar array. Fabricating a memristor crossbar needs much more complicated process steps than manufacturing memristors such as the isolated device. The number of measured memristors are around 50 in this paper. Actually, if we consider the memristor crossbar with a selector, as shown in Figure 2c, we can think the dynamic behavior measured from the isolated memristor can predict the dynamic behavior of the memristor crossbar very accurately because the selector can minimize the non-ideal effects such as sneak leakage, as shown in the recent experiment [29]. Additionally, in the network simulation, we considered various parasitic effects such as source resistance, neuron resistance, and wire resistance, as shown in Figure 5b [39,40,41]. By doing so, the non-ideal effects in the real fabricated crossbar could be taken into account by simulating the network’s accuracy, as shown in Figure 5, in this paper. Though we could not fabricate and measure the real memristor crossbar due to the complicated fabrication steps, we think that the measurement and simulation performed in this paper can validate the proposed asymmetrical scheme that is useful in saving the programming time and power in training the binary-crossbar-based neural networks.

In addition to the non-ideal effects such as source, neuron, and wire resistance, the other effects generated from inside the memristor devices cause the read-and-write noise when reading and updating the synaptic weights of memristor-crossbar networks [49]. They are thermal noise, random telegraph noise, wear-out mechanisms, etc., [49]. These noises can degrade the programming accuracy of memristor synapses when compared to the accuracy that we expected before without considering these memristor noises [49].

## 5. Conclusions

For realizing neural networks with binary memristor crossbars, memristors should be programmed by HRS and LRS, according to the training algorithms such as the backpropagation. Unfortunately, it takes a very long time and consumes a large amount of power in training memristor crossbars, because the program-verify scheme of memristors is based on the incremental pulse steps, where many programming and verifying pulses are repeated over until reaching to the target conductance. Thus, reducing the programming time and power during the crossbar training time is very essential for energy-efficient and fast training of memristor-based neural networks. In this paper, we compared four different programming schemes, which are F-F, C-F, F-C, and C-C, respectively. C-C means both HRS and LRS are programmed coarsely. C-F has the coarse-programmed HRS and the fine-programmed LRS, respectively. F-C is vice versa of C-F. In F-F, both HRS and LRS are fine-programmed. In terms of the product of the recognition error and training energy, C-F shows the best energy-efficiency among the four schemes. It means that the minimum error is achieved with the minimum energy consumption for C-F. The asymmetrical coarse-programmed HRS and fine-programmed LRS could reduce the programming time and energy during the training time of memristor crossbars because only LRS should be fine-programmed, while HRS is coarse-programmed. Moreover, the asymmetrical C-F can maintain the network’s error as low as F-F, due to the fact that the coarse-programmed HRS slightly degrades the recognition error. This energy-efficient and fast training of memristor-crossbar-based networks can be very useful in the nanoscale edge-computing systems, which demand the low-energy consumption desperately to process the vast amount of unstructured data from the physical environment.
